# Quantifiable Metrics for Predicting MSC Therapeutic Efficacy

**DOI:** 10.4172/2157-7633.1000365

**Published:** 2016-11-21

**Authors:** Siddaraju V Boregowda, Donald G Phinney

**Affiliations:** Department of Molecular Therapeutics, The Scripps Research Institute-Scripps Florida, USA

**Keywords:** Mesenchymal stromal cells, Mesenchymal stem cells, Clinical trials, Cellular therapy

## Introduction

Currently there are over 600 clinical trials listed at www. clinicaltrials.gov utilizing Mesenchymal Stromal Cells (MSCs) as an experimental cell-based therapy, making MSCs the most commonly employed cell type under investigation for treating human diseases. MSCs have gained widespread use in regenerative medicine due to their demonstrated potency in a broad range of experimental animal models of disease and their excellent safety profile in human clinical trials. However, while MSC-based therapies have clearly shown benefits in patients with ischemic and immune-related disorders, many trials completed to date have yielded suboptimal outcomes and several have failed to meet their primary endpoints of efficacy. A challenge in the development of efficacious MSC-based therapies is the inability to consistently manufacture homogeneous populations of cells with known efficacy for a specific disease indication that yield predictable and reproducible patient outcomes. This difficulty stems from the fact that methods routinely used to isolate MSCs [1–4] yield populations that exhibit significant heterogeneity in terms of morphologic features, growth rate, life span, differentiation potential, and potency in functional-based assays [5–8]. Donor-to-donor heterogeneity coupled with the lack of standardized manufacturing protocols makes it impossible to determine if patients enrolled in different clinical trials received functionally equivalent MSC preparations. The lack of metrics that discriminate functional differences between populations further confounds efforts to select the most suitable populations for a given disease indication. Therefore, the identification and reduction to practice of manufacturing schemes with deployable metrics to assess efficacy prior to patient administration is necessary to improve clinical outcomes and advance MSC-based therapies beyond the experimental phase.

## Univariate and Multi-variate Assays to Predict Potency of MSC Isolates

To achieve this goal, efforts are being focused on redefining MSC products based on their potency in biological assays rather than their phenotypic characteristics and/or composition of matter. For example, Lehman et al. [9] described a potency assay to quantify the pro-angiogenic activity of clinical grade MSCs (MultiStem ^®^) based on the ability of conditioned media to induce endothelial tube formation. This activity was shown to correlate with secreted levels of CXCL5, IL-8, and VEGF thereby allowing analysis of cytokines in spent media from manufacturing production runs to serve as a surrogate potency assay with pass/fail criteria. Similarly, Bloom et al. [10] quantified the ability of gamma irradiated MSCs to suppress T-cell proliferation at various effector-to-target cell ratios and used this information to calculate a mean suppression value for each isolate. While the immuno-potency assay had a broad dynamic range (27%–88%) it did not correlate with cell viability or HLA-DR expression levels. Using a sterile inflammation model of corneal injury, Lee et al. [11] demonstrated that human MSC populations expressing high TSG6 levels were most effective in reducing myeloperoxidase activity in the injured cornea. These data suggest that TSG6 may be a useful maker to identify MSC isolates for treating acute inflammatory diseases.

In cases where identifiable biomarkers are not available, more complicated multivariate analyses may be needed to determine potency. For example, while it is well accepted that MSCs possess osteogenic potential, the use of well-established bone-specific genes as biomarkers to predict this potential has proven unreliable [12–14]. To circumvent this problem, Murgia et al. [15] employed agglomerative cluster analysis of gene expression data to identify a subset of five genes that were consistently induced in MSC isolates after osteogenic induction in cell-based asays, and positively correlated with ectopic bone forming capacity as measured *in vivo*. A recent perspective from the International Society of Cellular Therapy (ISCT) also proposed use of a matrix-based approach employing gene and protein expression data coupled with functional-based assays with appropriate responder cells to evaluate the immuno-modulatory activity of MSC products used in clinical trials targeting immune-related disorders [16]. These studies serve as a blueprint for future assay development in cases where biomarkers are unknown or single cell-based assays are not sufficient to accurately model potency in human disease.

## Manipulation of MSCs to Enhance Efficacy Predictability

A significant amount of work has also been devoted to identifying culture conditions that augment or induce a specific functional trait in MSCs that may be exploited clinically. For example, Waterman et al. [17] demonstrated that human MSCs can be polarized by Toll-Like Receptor (TLR) engagment into two functionally distinct populations referred to as MSC1 and MSC2. In this scenario, TLR4-primed MSCs (MSC1) were shown to express pro-inflammtory mediators whereas TLR3-primed MSCs (MSC2) were shown to express immunosuppressive mediators. Furthermore, MSC1 cells failed to suppress T cell proliferation in mixed lymphocyte cultures whereas both unprimed MSCs and MSC2 cells were potently suppressive. Various groups have also shown that the immuno-suppressive activity of MSCs is induced in response to exposure to IFN-gamma [18,19] and Jin et al. [20] recently reported that IFN-gamma and TNF function synergistically to uniformly polarize MSCs toward a Th1 phenotype characterized by expression of the immunosuppressive factors IL-4, IL-10, CD274/PD-L1 and Indoleamine 2,3 Dioxygenase (IDO). Whether TLR primed or INF-gamma stimulated MSCs yield improved outcomes in clinical trials awaits further testing.

## A Posteriori Assay Development

While the aforemetioned studies represent signficant progress in the development of quantifiable assays to predict the therapeutic efficacy of MSC products, they suffer from several limitations. First, none of these assays attempt to link stem/progenitor and effector functions of MSCs. Second, they are limited to improving MSC efficacy in only one class of disease, e.g. inflammatory, ischemic or immune-related disorders. Third, each approach seeks to either improve efficacy or predict potency but not simulataneously achieve both results.

Recently, our laboratory performed a comparative anlaysis of multiple human MSC isolates to identify mechanisms that confer inter-population heterogeneity, and found that expressed levels of the transcription factor TWIST1 correlated with growth rate, survival, Colony Forming Unit-Fibroblast (CFU-F) activity, tri-lineage differentiation potential, angiogenic, anti-inflammatory, and immuno-modulatory activity [21]. Furthermore, we discovered that some traits were mutually exclusive in MSCs and that their gain/loss could be modeled via a hierarchical process. For example, we showed that MSCs characterized by rapid growth, high CFU-F activity, and a pro-angiogenic phenotype expressed high TWIST1 levels. Moreover, TWIST1 down regulation conferred an anti-inflammatory and immune-modulatory phenotype onto cells at the expense of angiogenic activity. Down regulation of TWIST1 also induced competence to undergo stimulus driven tri-lineage differentiation. Consistent with these findings, we demonstrated that manipulating TWIST1 levels predictably altered the pro-angiogenic and immune-modulatory activity of MSCs as quantified in cell-based assays as well as their therapeutic efficacy in a mouse model of acute lung injury. Together these findings demonstrate a direct mechanistic link between stem/progenitor and effector functions in MSCs and reveal that changes in these properties follows a hierarchical process that is dependent on TWIST1.

The fact that TWIST1 appears to play such a prominent role in dictating MSC fate and function is consistent with its known role in mesoderm specification and differentiation. For example, the protein was initially discovered in a mutagenesis screen as essential for mesoderm specification in Drosophila. [22] In rodents, TWIST1 activity is required for proper migration of neural crest cells in the first branchial arch and their proper differentiation into bone, muscle, and teeth [23]. Additionally, TWIST1 expression has been shown to be necessary for mesodermal cells including cranial mesoderm to maintain their mesenchymal characteristics [24]. The fact that some fraction of marrow-resident MSCs are derived from the neural crest [25,26] implicates TWIST1 in modulating their fate determination and self-maintenance.

Similarly, many of the salient features of our proposed MSC hierarchy are substantiated by previously published studies. For example, owing to the fact that HIF1α is a potent inducer of TWIST1, our data provide a mechanism by which hypoxic preconditioning potentiates the angiogenic activity of MSCs. Furthermore, studies that pre-condition MSCs with hypoxia, FGF2, or VEGF to enhance their angiogenic potential also report positive effects on cell growth, survival, and CFU-F. Conversely, treatment of MSCs with IFNG, which induces immune-suppressive activity, also downregulates TWIST1 resulting in impaired growth and reduced CFU-F activity. This result is consistent with the fact that interferons produce growth repressive effects by inhibiting expression of RPL23A [27], which is also a TWIST1 target in MSCs. Other studies have shown that differentiation of MSCs to the osteoblast lineage results in a concomitant up regulation of IFNG-inducible genes [28,29] while impairing angiogenic activity [30]. Together, these findings provide additional experimental support for the proposed MSC hierarchy.

## A Clinical Indications Prediction (CLIP) Scale to Assess MSC Potency

**Figure 1:**
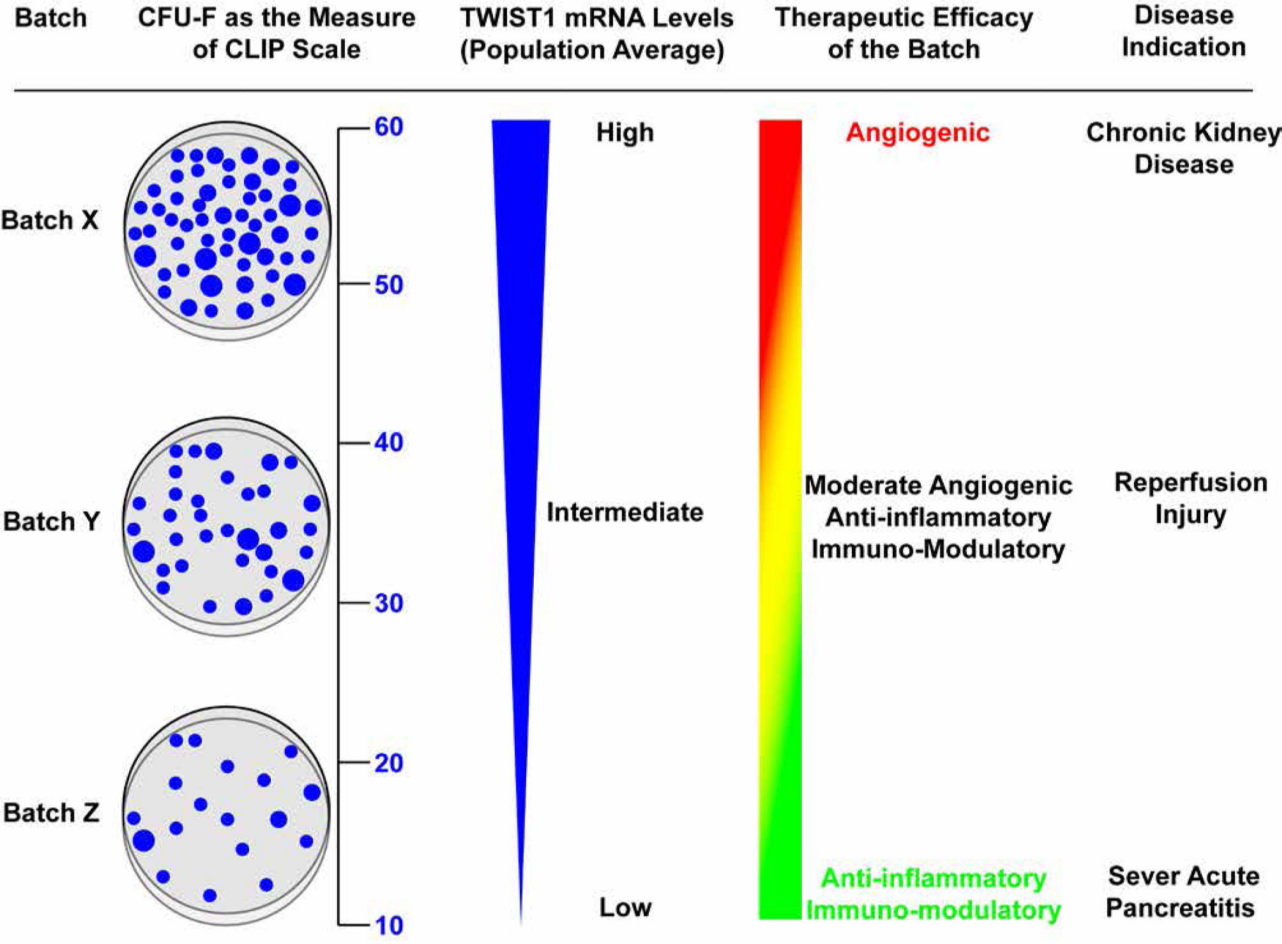
Schematic illustrating how the CFU-F assay can be adapted across labs to estimate TWIST1 levels in human MSC isolates, which serves as a predictor of therapeutic efficacy. Viable cells (100) from a patient-ready batch of MSCs are sorted by flow cytometry and deposited into a 10-cm dish then cultured for 10 days. Based on the number of CFU-Fs (numerical scale, left) the batch will be designated as exhibiting high, intermediate, or low TWIST1 levels, and its predicted therapeutic efficacy is then determined based on the CLIP scale. For example, MSC batch X that gives >50 CFU-Fs will express high TWIST1 levels and therefore be assigned with ischemic disease indications like chronic kidney disease for regeneration of capillaries in the glomerulus. MSC batch Z that gives <20 CFU-Fs will express low TWIST1 levels and be assigned to immune and acute inflammatory disease indications like sever acute pancreatitis where suppression of acute inflammation is beneficial. MSC batch Y that gives 20–50 CFU-Fs will express intermediate TWIST1 levels and be assigned to disease indications like ischemic reperfusion injury where both moderate angiogenic and anti-inflammatory activity of MSCs is beneficial.

Based on the aforementioned studies, we developed a CLinical Indications Prediction (CLIP) scale that predicts the therapeutic efficacy of different human MSC isolates for a given disease indication based on TWIST1 expression levels, which specifies the position of cells within the established hierarchy [21]. The CLIP scale is advantageous over other potency assays as it predicts differences in growth, survival, stem/progentior, and effector functions of MSCs rather than just a single function, and can easily be correlated to quantifiable functional assays. For example, we reported that TWIST1 levels are positively correlated with CFU-F activity. Therefore, use of a standard CFU-F assay may provide a simple and reproducible functional assay to place a given MSC batch on the CLIP scale (Figure 1) to assess efficacy.

Unlike small molecule drugs where producing a ‘batch’ is possible, MSC-based therapies incorporate a multitude of donor populations, culture conditions, and culture supplements to produce lots for clinical dosing. Therefore, establishing a ‘batch” of MSCs for use in a broad array of diseases poses a unique challenge for which there is no precedence in drug discovery. The CLIP scale attempts to remedy this problem. For example, since TWIST1 levels directly regulate CFU-F, any variations in culture conditions including lots of bovine sera, platelet lysate, proprietary formulations of animal-free media, and growth factor supplementation, as well as cell passage number may be accounted for by the CFU-F assay. Therefore, the CLIP scale can negate the need for using a universal standard manufacturing platform and a universal MSC standard as a metric for comparison. Most importantly, by further elucidating the mechanisms responsible for establishing and maintaining the MSC hierarchy, additional functional metrics can be incorporated into the CLIP scale to expand its usefulness and robustness in predicting the therapeutic efficacy of clinical grade MSC lots for different disease indications. This approach would result in improved patient outcomes in future clinical trials.
